# Coexistence of 3G Repeaters with LTE Base Stations

**DOI:** 10.1155/2013/185372

**Published:** 2013-12-29

**Authors:** Woon-Young Yeo, Sang-Min Lee, Gyung-Ho Hwang, Jae-Hoon Kim

**Affiliations:** ^1^Department of Information and Communication Engineering, Sejong University, 98 Gunja-dong, Gwangjin-gu, Seoul 143-747, Republic of Korea; ^2^Network Technology R&D Center, SK Telecom, 9-1 Sunae-dong, Bundang-gu, Seongnam 463-838, Republic of Korea; ^3^Department of Computer Engineering, Hanbat National University, San 16-1 Dukmyung-dong, Yuseong-gu, Daejeon 305-719, Republic of Korea; ^4^Department of Industrial Engineering, Ajou University, Yeongtong-gu, Suwon 443-749, Republic of Korea

## Abstract

Repeaters have been an attractive solution for mobile operators to upgrade their wireless networks at low cost and to extend network coverage effectively. Since the first LTE commercial deployment in 2009, many mobile operators have launched LTE networks by upgrading their 3G and legacy networks. Because all 3G frequency bands are shared with the frequency bands for LTE deployment and 3G mobile operators have an enormous number of repeaters, reusing 3G repeaters in LTE networks is definitely a practical and cost-efficient solution. However, 3G repeaters usually do not support spatial multiplexing with multiple antennas, and thus it is difficult to reuse them directly in LTE networks. In order to support spatial multiplexing of LTE, the role of 3G repeaters should be replaced with small LTE base stations or MIMO-capable repeaters. In this paper, a repeater network is proposed to reuse 3G repeaters in LTE deployment while still supporting multilayer transmission of LTE. Interestingly, the proposed network has a higher cluster throughput than an LTE network with MIMO-capable repeaters.

## 1. Introduction

Long Term Evolution (LTE) is a radio platform that allows mobile operators to achieve much higher peak data rates and better spectral efficiency than those of the third generation (3G) networks (e.g., WCDMA and cdma2000) [1]. LTE was initiated by the Third Generation Partnership Project (3GPP) in 2004 and is now commercially deployed or in progress worldwide. LTE is an OFDM-based, radio-access technology that supports scalable bandwidth up to 20 MHz and multiple-input multiple-output (MIMO) transmission with up to four antennas. It provides high peak data rates, with a potential for 300 Mbps on the downlink by using 20 MHz and four transmit antennas. LTE was further improved to LTE-Advanced, which was standardized in 2010 as part of 3GPP Release 10 features [2]. LTE-Advanced is not defined as new specification series but built on existing LTE specifications.

As in other mobile communication systems, cell planning for an LTE network is focused on extending LTE coverage at minimum cost. Repeaters permit mobile operators to upgrade their 3G and legacy networks to LTE at low cost and to extend LTE coverage effectively. Because most of 3G mobile operators will eventually upgrade their networks to LTE and existing 3G frequency bands are shared with the frequency bands identified for LTE deployment [3, 4], reusing 3G repeaters in the LTE network may be a practical and cost-efficient solution. However, it is difficult to use the 3G repeaters directly in the LTE network because LTE requires MIMO transmission with multiple antennas. To support MIMO transmission, repeaters should also support the same number of antenna ports as a base station (BS). If repeaters are not MIMO-capable, they can act as a keyhole, and the channel rank in repeater coverage is reduced to one [5]. Because mobile operators have an enormous number of 3G repeaters for their own 3G networks, high investment costs are required to replace 3G repeaters with small LTE BSs or MIMO-capable repeaters. In this paper, a repeater network is proposed to reuse 3G repeaters instead of the high-cost equipment and take advantage of multiantenna transmission of LTE.

## 2. Multiantenna Transmission

**Figure 1:**
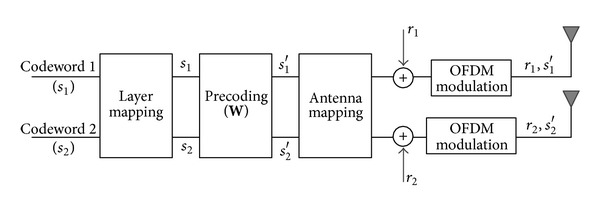
Basic structure of LTE multiantenna transmission (2 × 2 antenna configuration).

The use of multiple antennas is one of the key technologies to achieve LTE performance targets [6]. Multiantenna transmission in LTE can be described as a mapping of modulated symbols to different antenna ports.  Figure 1 illustrates downlink multiantenna transmission that supports spatial multiplexing of up to two layers in a 2 × 2 antenna configuration. A codeword is an independently encoded data block, corresponding to a single transport block (TB) delivered from an L2 protocol. A spatial layer is one of the different streams generated for spatial multiplexing and layer mapping is a process of distributing codewords over multiple spatial layers based on the number of antenna ports. Precoding can be used to improve signal isolation at the receiver side and provide mapping of the spatially multiplexed streams to transmit antennas (including beam-forming). The precoded symbols are mapped to appropriate antenna ports by antenna mapping and reference symbols (*r*_1_ and *r*_2_) are inserted within an OFDM time-frequency grid.

The number of layers may range from a minimum of one up to multiple layers. The number of layers is often referred to as the transmission rank and LTE can adaptively control the transmission rank according to channel conditions, such as received signal quality and fading correlation between antennas. In a 2 × 2 antenna configuration, there are four 2 × 1 precoding matrices for rank-1 transmission and three 2 × 2 matrices for rank-2 transmission as shown in Table 1 [7]. Codebook index 0 for rank 2 (i.e., identity matrix) is not used in a closed-loop operation but only applied to an open-loop operation. When closed-loop precoding is configured, each user equipment (UE) can report a suitable transmission rank by rank indication (RI) and a precoding matrix by precoding matrix indication (PMI) based on reference signal measurements. However, RI and PMI are only recommendations and the BS does not have to follow the RI/PMI provided by the UE.

In rank-2 transmission with a 2 × 2 antenna configuration, data symbols (*s*_1_ and *s*_2_) from two different codewords are mapped to the first and second layers, respectively. If a UE does not support rank-2 transmission due to poor channel quality, rank 1 can be used to improve the SINR by diversity gains. In the case of rank-1 transmission, only one codeword (*s*_1_) is mapped to a single layer, and one of the precoding matrices for rank 1 is applied to adjust antenna weighting. Letting **y** be the received signal on each subcarrier at the UE, a general transmission model is described by

(1) y=[h11h12h21h22][s1′s2′]+[n1n2]={HWs1+n,for  rank⁡  1HW[s1s2]+n,for  rank⁡  2,

where *h*_*ij*_ denotes the channel fading coefficient between the *i*th receive antenna and the *j*th transmit antenna, *s*_1_′ and *s*_2_′ are output symbols after precoding, and *n*_*i*_ is the combined noise and interference at the *i*th receive antenna. **H** is the 2 × 2 channel matrix, **W** is the precoding matrix in Table 1, and **n** represents the combined noise and interference vector at the UE. In order to detect and receive wanted signals at the receiver, linear and nonlinear algorithms can be used depending on implementation and complexity of the receiver [8].

In LTE, the control region in a subframe (1 ms) carries L1/L2 signaling necessary to control uplink and downlink data transmission, and transmit diversity is generally applied to the signaling [1]. In a 2 × 2 antenna configuration, the transmit diversity is based on Space-Frequency Block Coding (SFBC). Two consecutive modulation symbols, *s*_1_ and *s*_2_, are mapped directly to the *k*th and (*k* + 1)th subcarriers, respectively, on the first antenna port. On the second antenna port, −*s*_2_* and *s*_1_* are mapped to the corresponding subcarriers. The received SFBC signals on the *k*th and (*k* + 1)th subcarriers, **y** ^(*k*)^ and **y** ^(*k*+1)^, respectively, are given by

(2) y(k)=[h11(k)h12(k)h21(k)h22(k)][s1−s2∗]+n(k),

(3) y(k+1)=[h11(k+1)h12(k+1)h21(k+1)h22(k+1)][s2s1∗]+n(k+1),

where *h*_*ij*_^(*k*)^ and **n** ^(*k*)^ have the same definitions as (1). Assuming that the MIMO channel is quasistatic (i.e., *h*_*ij*_^(*k*)^ ≈ *h*_*ij*_^(*k*+1)^), it is possible to use a simple decoder to achieve the frequency diversity of SFBC. In addition, the orthogonal design of SFBC can support maximum likelihood (ML) detection based on linear processing at the receiver [9].

## 3. Coexistence of 3G Repeaters in LTE Networks

Repeaters have been an essential building block of communication systems for a long time. The most significant advantages of repeaters are their fast deployment capability and cost efficiency. Typically, repeaters are implanted in the network optimization phase when network coverage needs to be improved. Hence, classical applications of repeaters are related to coverage enhancement that targets providing a strong signal to remote areas where wireless signal strength is relatively weak.

Generally, a repeater system consists of two parts: a donor unit and a remote unit. The repeater system transparently conveys and amplifies wireless signals between a BS and UEs. The donor unit captures a BS signal via a direct coupler near the BS and transmits the amplified signal to the remote unit, typically via an RF signal (RF repeater) or fiber optic cables (fiber optic repeater). The remote unit will reconvert the received signal into an RF signal and send the signal to repeater coverage. The uplink signal is also amplified and retransmitted to the BS in the opposite direction.

In 3GPP technical specifications, LTE is designed to operate in all the frequency bands defined for 3G networks [3, 4]. In FDD, bands 1–14, 19–22, and 25 can be applied to both LTE and 3G systems, and four additional operating bands are reserved for LTE deployment. Although other frequency bands can be undertaken depending on regional and local conditions, LTE will be deployed in the same frequency bands as 3G and other legacy cellular technologies. Moreover, most of 3G repeaters can support a wide working bandwidth (e.g., 20 MHz) that corresponds to multiple carriers of 3G systems. Thus, it is a reasonable, cost-efficient solution to reuse the 3G repeaters when upgrading 3G networks to LTE. However, there is a critical problem that 3G repeaters cannot coexist with LTE networks; conventional 3G repeaters have only one antenna port and usually do not support spatial multiplexing. In order to support the spatial multiplexing of LTE, the role of 3G repeaters should be replaced with small LTE base stations or MIMO-capable repeaters. Two of possible ways to reuse conventional 3G repeaters are (1) configuring rank-1 transmission over all service areas and (2) installing multiple 3G repeaters at a repeater site to support multiple antenna ports. The first method does not guarantee full capacity of the LTE system, and the second is unrealistic due to its excessive cost of reengineering.

The proposed repeater architecture is devised to overcome the problem of capacity degradation when conventional 3G repeaters are connected to the LTE BS. The basic idea of the proposed network is to extract multiple rank-1 data streams from the BS antenna ports and deliver each of them to one or more repeaters. Before introducing the proposed scheme, assume that, in rank-2 transmission with a 2 × 2 antenna configuration, each BS antenna port is simply connected to a separate repeater without precoding (i.e., **W** = **I**). Because one codeword for each spatial layer can be transmitted transparently to separate repeater coverage, it is expected that a UE in the repeater coverage can receive its data by a normal rank-1 operation. However, the UE may not receive the data because each antenna port contains only a single reference signal (RS) pattern for a specific spatial layer. For example, the second antenna port does not contain *r*_1_ (RS for antenna port 1), which is critical for rank-1 transmission. Basically, both of the two RS patterns (*r*_1_ and *r*_2_) are necessary to decode the data symbol when two transmit antenna ports are configured at the BS.

**Figure 2:**
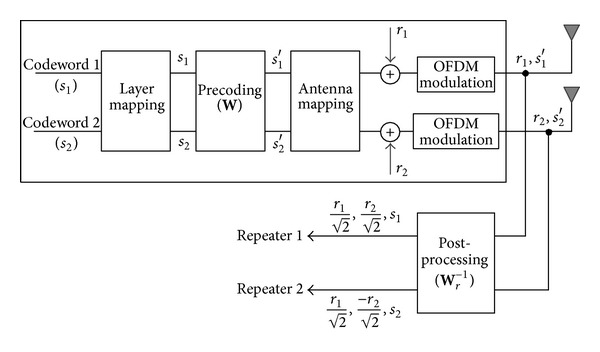
Proposed architecture supporting 3G repeaters.

The proposed architecture is illustrated in Figure 2 assuming a 2 × 2 antenna configuration, which is one of the main transmission configurations in the LTE network. Before being connected to each repeater, the BS signal is processed by an operation called post-processing, which is an inverse operation of precoding for repeaters and is expressed as **W**_*r*_^−1^ in Figure 2. Although there are three precoding matrices specified for rank 2 in Table 1, we set a precoding matrix for repeater coverage, **W**_*r*_, as follows:

(4) Wr=Wr−1=12[111−1].

Original data symbols (*s*_1_ and *s*_2_) can be extracted from *s*_1_′ and *s*_2_′ by the post-processing operation. In addition, due to the post-processing and mutually exclusive positions of RS, the first repeater path contains $r_{1}/\sqrt{2}$, $r_{2}/\sqrt{2}$, and *s*_1_, and the second path contains $r_{1}/\sqrt{2}$, $-r_{2}/\sqrt{2}$, and *s*_2_. Now, each repeater path contains all reference symbols and wanted data (*s*_1_ or *s*_2_), which are required for normal rank-1 transmission in a 2 × 2 antenna configuration. The post-processing by (4) can be understood as an addition of the two antenna ports for the first repeater path and a subtraction for the second path. The post-processing is a fixed operation, so that it is applied to all signals generated by the BS and has a meaning only to the UEs in repeater coverage. Note that the post-processing is performed at the end of OFDM modulation and does not modify the BS modem structure.

As for scheduling, the entire bandwidth is shared by a BS and the associated repeaters. The minimum unit of scheduling in LTE is a time-frequency block corresponding to one subframe and one resource block (RB, 180 kHz). If some RBs are reserved for the BS (or repeater) coverage, they cannot be allocated to the repeater (or BS) coverage. The BS scheduler should select an appropriate precoding matrix depending on the UE location. **W**_*r*_ in (4) is selected for the UEs in repeater coverage, whereas one of the precoding matrices in Table 1 is adaptively selected for the UEs in BS coverage. If the repeater coverage is selected for scheduling, two different codewords for the first and second repeater paths are provided to the layer mapping block. If there is no UE in one of the two repeater areas, just one codeword is transmitted over repeater coverage. In the proposed scheme, because an unwanted signal is also radiated in the unscheduled coverage, it can act as interference to the UEs in other coverage. In addition, the UE location needs to be known in advance to the BS scheduler because the transmission rank and precoding information may be different depending on the UE location. The UE location can be tracked by an uplink sounding reference signal (SRS), which is used by the BS for channel state estimation. After comparing the power levels from three receiving paths (one BS path and two repeater paths), the BS considers the service area having the highest SRS power level as the scheduling target for the UE.

Before proceeding to performance evaluation, it should be verified that UEs in repeater coverage have no problem in receiving data and control signals in the proposed scheme. As for data transmission, transmission mode 4 (TM-4, closed-loop codebook-based precoding) with rank-1 precoding is applied to the UEs in repeater coverage. The codebook indices 0 and 1 for rank 1 in Table 1 correspond to the column vectors of **W**_*r*_ in (4) and they are selected for the precoding information of the first and second repeater paths, respectively. Letting **y**_1_ and **y**_2_ denote the received signals at a UE in the first and second repeater coverage, respectively,

(5) y1=[h1h2]s1+n1

(6) =(12[h1h1h2h2])(12[11])s1+n1,

(7) y2=[h1h2]s2+n2

(8) =(12[h1−h1h2−h2])(12[1−1])s2+n2,

where *h*_*i*_ is the channel fading coefficient between a repeater and the *i*th receive antenna of the UE and **n**_*j*_ represents the combined noise and interference vector in the *j*th repeater coverage. The received signal from a repeater is simply expressed as (5) and (7), and each can be decomposed into the product of a channel matrix and a precoding matrix as in (6) and (8). The channel coefficients in (6) and (8) are obtained by measuring the RS in repeater coverage. Note that the original reference symbols are scaled down by a factor of $\sqrt{2}$ in the repeater coverage due to the post-processing. It is interesting that the inverted RS estimation for the second repeater coverage in (8) is compensated by codebook index 1 for rank 1 in Table 1. Because the received signal at the UE has the same form as (1) for rank-1 transmission, data symbols can be recovered by a normal rank-1 operation of TM-4. Now, assuming the TM-4 operation supporting rank adaptation in BS coverage, TM-4 can be applied to all UEs in the proposed network regardless of the UE location.

In the case of the downlink L1/L2 signaling, control symbols should be received correctly even in the repeater coverage. Letting **y**_1_^(*k*)^ and **y**_2_^(*k*)^ denote the received SFBC signals on subcarrier *k* in the first and second repeater coverage, respectively, the received control signals can be expressed as follows:

(9) y1(k)=s1−s2∗2[h1(k)h2(k)]+n1(k)

(10) =(12[h1(k)h1(k)h2(k)h2(k)])[s1−s2∗]+n1(k),

(11) y1(k+1)=s2+s1∗2[h1(k+1)h2(k+1)]+n1(k+1)

(12) =(12[h1(k+1)h1(k+1)h2(k+1)h2(k+1)])[s2s1∗]+n1(k+1),

(13) y2(k)=s1+s2∗2[h1(k)h2(k)]+n2(k)

(14) =(12[h1(k)−h1(k)h2(k)−h2(k)])[s1−s2∗]+n2(k),

(15) y2(k+1)=s2−s1∗2[h1(k+1)h2(k+1)]+n2(k+1)

(16) =(12[h1(k+1)−h1(k+1)h2(k+1)−h2(k+1)])[s2s1∗]+n2(k+1),

where *h*_*i*_^(*k*)^, **n**_1_^(*k*)^, and **n**_2_^(*k*)^ have the same definitions as (5)–(8). Note that precoding is not applied to transmit diversity of SFBC. The transmitted symbols from the first and second repeaters are addition and subtraction of the two SFBC symbols, respectively, on each subcarrier, scaled down by a factor of $\sqrt{2}$. The channel coefficients in (10), (12), (14), and (16) are obtained by measuring the RS in repeater coverage as explained in data symbol decoding. Because the received SFBC signal from a repeater can be expressed as a normal SFBC signal in (2) and (3), the control symbols in repeater coverage can be recovered by the same estimation technique as in the BS coverage. Now, we can see that all UEs in the proposed network can receive downlink signaling regardless of the UE location.

## 4. Results

**Figure 3:**
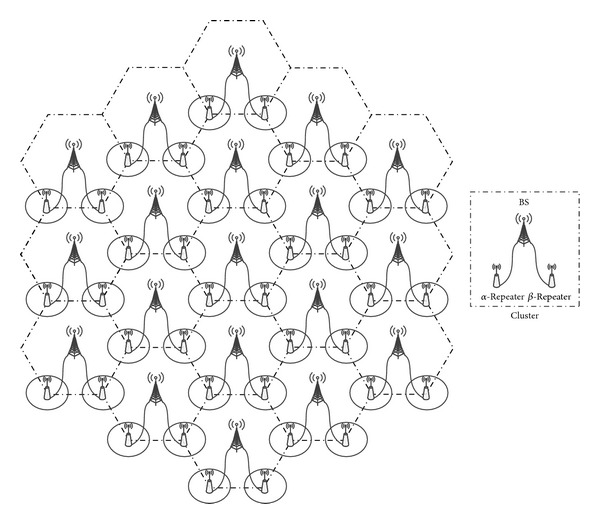
Cell layout for performance evaluation. The concept of *α* and *β* repeaters is used only in the proposed scheme. In other repeater networks, a single type of repeater is connected to the BS.

The simulation framework is based on the Vienna LTE system level simulator [10] and includes additional procedures and algorithms used in the proposed scheme. We consider a basic hexagonal layout composed of BSs and repeaters.  Figure 3 shows the network layout for the simulation. The BSs and repeaters are located at the center and vertices of the hexagonal area, respectively, with an omni-directional antenna (or antennas). A cluster consists of one BS and two repeaters connected to the BS. 18 clusters are added around the central cluster and statistics are only collected from the central cluster. In the proposed scheme, one repeater in a cluster is connected to the first repeater path (*α*) and the other repeater to the second path (*β*). In other conventional repeater networks, a single type of repeater is connected to the BS and transmits the same signal as the BS. 3G repeaters have one transmit antenna. BSs and MIMO-capable repeaters have two transmit antennas and they support spatial multiplexing with rank adaptation. UEs have two receive antennas.

The main simulation parameters are summarized in Table 2. The path loss is based on an urban macrocell with a carrier frequency of 2 GHz and a BS antenna height of 15 m above average rooftop level [11]. Shadowing is not considered in this simulation and the microscale fading uses a pedestrian A channel at 3 km/h. The distance between a BS and neighboring repeaters is 1,000 m. A system bandwidth of 10 MHz is assumed, which corresponds to 50 RBs. The BS transmits at full power (*P*_*b*_ = 40 W) over the entire bandwidth. The repeater power (*P*_*r*_) is usually lower than the BS power, and some typical power levels will be selected for repeaters. We assume two OFDM symbols for the control region in a subframe, with a normal cyclic prefix (CP) configuration. MIMO modeling is based on a zero forcing (ZF) receiver. The level of interference may be different depending on the operation modes of neighboring nodes. For example, the intercell interference caused by rank-2 transmission is slightly higher than that by rank-1 transmission, based on the channel model in [10]. Thus, in this simulation, the surrounding network nodes are assumed to be of rank 1 and rank 2 with probability 0.5 and 0.5, respectively, if they support rank adaptation (i.e., LTE BSs and MIMO-capable repeaters).

The scheduling policy is a proportional fair (PF) scheduler, which maximizes the total throughput while offering certain fairness among UEs. The UE with the highest *p* value (ratio of instantaneous data rate to average data rate) is selected for each RB. In the proposed scheme, the BS scheduler compares the *p* value for the BS coverage with the sum of the two *p* values for the *α* and *β* repeaters. The BS scheduler selects the coverage with a higher value. When the repeater coverage is selected by the scheduler, the corresponding RB is shared by two UEs having the highest *p* value in each repeater area. If there is no UE in one of the two repeater areas, the scheduler selects just one UE having the highest *p* value in the other repeater area.

Because the OFDM-based repeater network can be considered a simplified form of a single-frequency network (SFN), all signals that arrive at the receiver within a CP are treated as useful [12]. In this simulation, the repeater network is assumed to be well engineered, so that all downlink signals in a cluster arrive at the UE within the CP. However, in the proposed scheme, the received signals from the BS, *α*-repeater and *β*-repeater, can interfere with one another even if they arrive within the CP.

**Figure 4:**
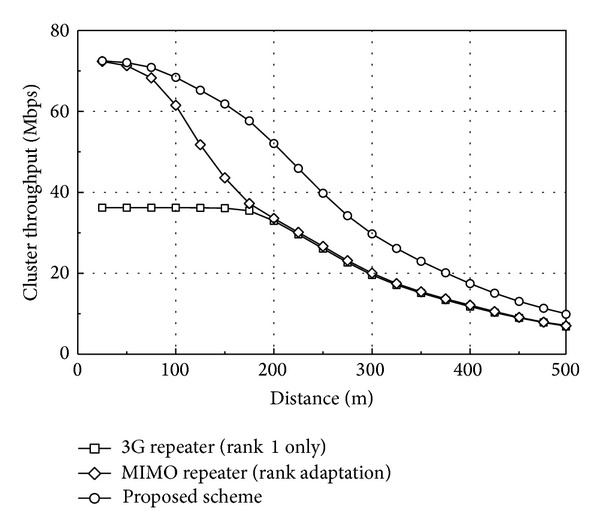
Cluster throughput at different distances. Two UEs are located at the same distance from the BS (or repeater) in each coverage (six UEs per cluster).

Figure 4 compares the cluster throughput as a function of distance from the BS (or repeater). The repeaters transmit the same power as the BS (*P*_*r*_ = 40 W) and two UEs in each coverage are located at the same distance from the BS (or repeater). As the distance increases, the cluster throughput decreases due to the degradation of signal quality. The MIMO-capable repeater produces a higher throughput than the 3G repeater does; however, the difference becomes smaller as the distance increases. The reason is that only the UEs near the BS (or repeater) can support rank-2 transmission and the proportion of rank-2 transmission decreases rapidly in the MIMO-capable repeater network. At a distance of 200 m, 20% of RI reported from the UEs is classified as rank 2, and the aggregate TB size of rank-2 transmission is only slightly larger than that of rank-1 transmission. The situation becomes even worse at 250 m, where only 5% of RI is classified as rank 2.

The proposed scheme is the best among the three repeater networks. The cluster throughput is higher than other repeater networks even if the distance increases. The reason is that the proposed scheme can be considered a similar form of multiuser MIMO transmission over repeater coverage. When repeater coverage is selected by the BS scheduler, two different data streams are transmitted simultaneously in the *α* and *β* repeater areas, respectively, guaranteeing rank-2 transmission from the BS point of view. As the distance increases, the proportion of rank-2 transmission decreases rapidly in the MIMO-capable repeater network, but the proposed scheme can still support dual-stream transmission by means of scheduling over repeater coverage. The ratio of selecting repeater coverage in the proposed scheme is about 66% regardless of the distance. Thus, the performance of the proposed scheme can outperform the MIMO-capable repeater network with rank adaptation. The disadvantage of the proposed scheme is that the peak data rate in the repeater coverage can be half of that in the BS coverage.

**Figure 5:**
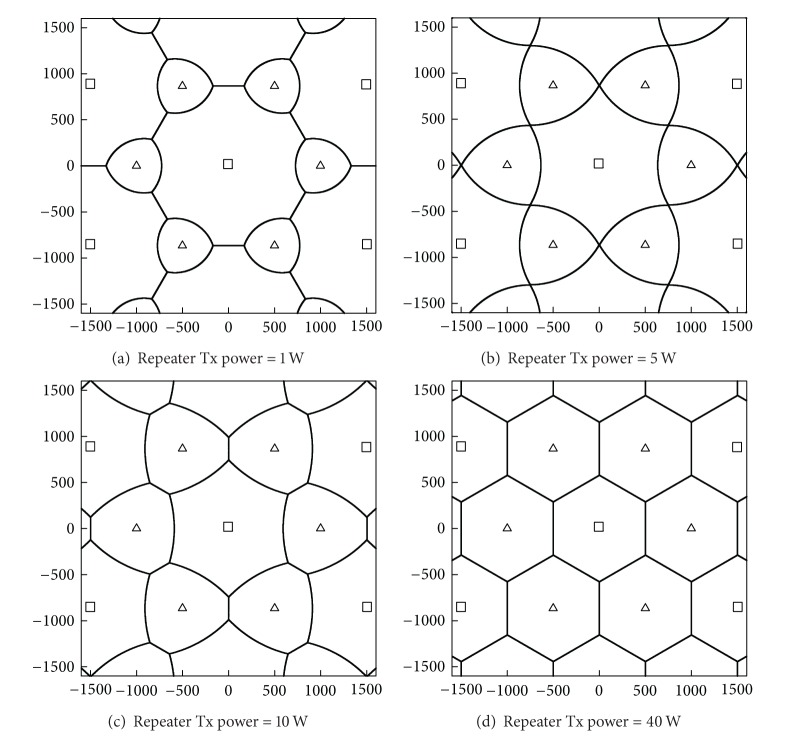
Coverage boundaries of base stations and repeaters with different repeater power levels. Squares and triangles represent base stations and repeaters, respectively. Units of *x*- and *y*-axes are in meters.

Because the repeater power is usually lower than the BS power, it is reasonable to take the repeater power into account for performance evaluation.  Figure 5 illustrates coverage boundaries when *P*_*r*_ is set to 1, 5, 10, and 40 W, respectively. As expected, at a low value of *P*_*r*_, the repeater coverage is relatively small when compared to the BS coverage. Accordingly, only a small number of UEs will be located in the repeater coverage if the UEs are uniformly distributed. If the repeater power is the same as the BS power, the coverage boundary becomes a typical hexagonal structure.

**Figure 6:**
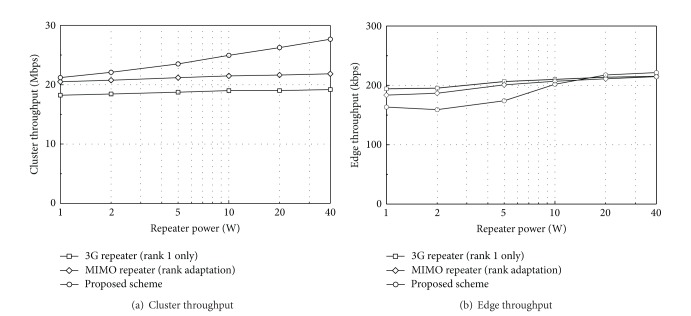
Performance comparison at different transmission power of repeaters.

Figure 6 compares the cluster throughput and the edge throughput of the three repeater networks when 30 UEs are randomly distributed in each cluster. 1,000 different UE distribution scenarios are applied to the simulation. In Figure 6(a), as the repeater power increases, the cluster throughput increases steadily in the proposed scheme. More UEs will be located in repeater coverage at a higher value of *P*_*r*_. Remember that two data streams can be transmitted simultaneously over repeater coverage in the proposed scheme when repeater coverage is selected for scheduling. As the repeater coverage increases, the ratio of scheduling on the repeater coverage also increases. For example, the ratio increases from 18.1% to 64.1% as the repeater power increases from 1 W to 40 W. As a result, at *P*_*r*_ = 40 W, the cluster throughput of the proposed scheme is 26.7% higher than that of the MIMO-capable repeater network. It is interesting that the MIMO-capable repeater network has just 13% higher throughput than the 3G repeater network. Only about 13.5% of RI is classified as rank 1 in the MIMO-capable repeater network, and thus the cluster throughput improvement is not as significant as expected.

In Figure 6(b), the edge throughput is defined as the data rate that 5% of the UEs cannot reach [11]. The edge throughput of the MIMO-capable repeater network is slightly lower than that of 3G repeater network. The reason is that the interference caused by rank-2 transmission is slightly higher than that by rank-1 transmission, as explained in simulation parameters. The edge throughput of the proposed scheme is rather lower than those of other repeater networks with *P*_*r*_ = 1, 2, and 5 W, whereas almost the same or slightly higher with *P*_*r*_ = 10, 20, and 40 W. This can be explained by two factors that influence the performance of the proposed scheme. The first factor is the increased interference compared to other repeater networks. The received signals from the BS, *α*-repeater and *β*-repeater, can interfere with one another in the proposed scheme because they are different signals. This additional interference can cause degradation of the edge throughput. The second factor is dual-stream scheduling over repeater coverage. When repeater coverage is selected for scheduling, two UEs connected to the *α* and *β* repeaters, respectively, can receive downlink data at the same time. This can give the UEs in repeater coverage the opportunity to receive more data, and thus the edge throughput can be improved. At low repeater power, because only a small number of UEs are located in repeater coverage, the first factor is dominant over the second one. At high repeater power, the dual-stream scheduling overcomes the interference on the edge UEs and the edge throughput becomes slightly higher than those of other networks. In summary, at repeater power over 10 W, the cluster throughput of the proposed scheme is much higher than those of other repeater networks while the edge throughput is almost the same or slightly higher.

## 5. Conclusions

In this paper, a repeater network has been proposed to reuse 3G repeaters in LTE deployment and utilize the multiantenna structure of LTE effectively. Because 3G repeaters usually do not support multiantenna transmission, the proposed scheme extracts two rank-1 data streams from the BS antenna ports and delivers each of them to one or more repeaters by a post-processing operation in the base station. It has been proved that the closed-loop codebook-based precoding and transmit diversity based on SFBC can be applied to all UEs in the proposed network. Due to the simultaneous transmissions of multiple data streams in repeater coverage, the proposed scheme showed a much higher throughput than a MIMO-capable repeater network while maintaining an acceptable level of edge throughput.

## Figures and Tables

**Table 1:** Precoding matrices (**W**) for two antenna ports.

Codebook index	0	1	2	3
Rank 1	$\frac{1}{\sqrt{2}}\left[\begin{array}{c} 1\\ 1 \end{array}\right]$	$\frac{1}{\sqrt{2}}\left[\begin{array}{c} 1\\ -1 \end{array}\right]$	$\frac{1}{\sqrt{2}}\left[\begin{array}{c} 1\\ j \end{array}\right]$	$\frac{1}{\sqrt{2}}\left[\begin{array}{c} 1\\ -j \end{array}\right]$

Rank 2	$\frac{1}{\sqrt{2}}\left[\begin{array}{cc} 1 & 0\\ 0 & 1 \end{array}\right]$	$\frac{1}{2}\left[\begin{array}{cc} 1 & 1\\ 1 & -1 \end{array}\right]$	$\frac{1}{2}\left[\begin{array}{cc} 1 & 1\\ j & -j \end{array}\right]$	—

**Table 2:** Simulation parameters.

Parameters	Values
Path loss model	128.1 + 37.6 log_10_⁡(*x*), *x* in km
Microscale fading	Pedestrian A channel at 3 km/h
Intersite distance	1,000 m
System bandwidth	10 MHz (50 RBs)
BS power (*P*_*b*_)	40 W (fixed)
Repeater power (*P*_*r*_)	1 to 40 W (variable)
MIMO receiver modeling	Zero forcing
Traffic generation	Full buffer
Scheduling policy	Proportional fair
UE noise figure	9 dB
Thermal noise	−174 dBm/Hz
